# {(*R*,*S*_Fc_,*S*_Fc_)-2′′-Bromo-2-[1-(dimethyl­amino)­ethyl-κ*N*]-1,1′′-biferrocene}trihydridoboron

**DOI:** 10.1107/S1600536811049270

**Published:** 2011-11-23

**Authors:** Yaping Wang, Afrooz Zirakzadeh, Walter Weissensteiner, Kurt Mereiter

**Affiliations:** a Capital Medical University, Beijing, No. 10 Xitoutiao, You An Men Beijing 100069, People’s Republic of China; b Institute of Organic Chemistry, University of Vienna, Währingerstrasse 38, A-1090 Vienna, Austria; c Institute of Chemical Technologies and Analytics, Vienna University of Technology, Getreidemarkt 9/164SC, A-1060 Vienna, Austria

## Abstract

The title structure, [Fe_2_(C_5_H_5_)_2_(C_14_H_19_BBrN)], contains a chiral and asymmetrically 2,2′′-disubstituted biferrocene designed as precursor for enanti­oselective non-*C*_2_-symmetric biferrocenyldiphosphine catalysts. The mean bond lengths in the biferrocene unit are Fe—C = 2.048 (10) Å and C—C = 1.427 (8) Å within the cyclo­penta­dienyl rings. The B—N bond lengths of the BH_3_ protected amine is 1.631 (3) Å. The inter­planar angle between the two connected cyclo­penta­dienyl rings is 54.29 (8)° and the corresponding Fe—*Cg*—*Cg*—Fe torsion angle is −52.5°. The conformation of the mol­ecule is stabilized by an intra­molecular C—H⋯Br inter­action.

## Related literature

For general information on ferrocene-based diphosphines and their applications in asymmetric catalysis, see: Togni (1996 ▶); Blaser *et al.* (2007 ▶); Dai & Hou (2010 ▶). For the synthesis, coordination behavior and use in asymmetric catalysis of ligands based on biferrocenes, see: Sawamura *et al.* (1991 ▶); Nettekoven *et al.* (2000 ▶); Xiao *et al.* (2002 ▶); Espino *et al.* (2009 ▶); Kuwano (2010 ▶). For synthetic aspects of the title compound, see: Wang *et al.* (2011 ▶).
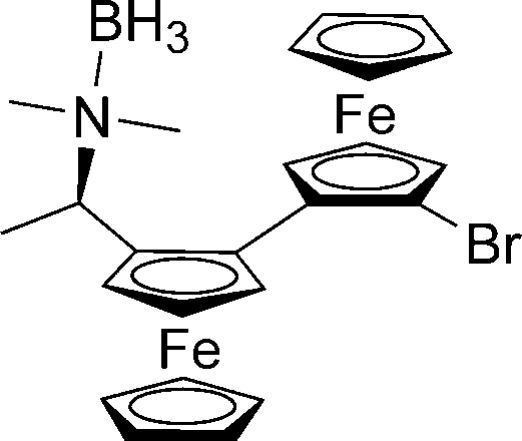


## Experimental

### 

#### Crystal data

[Fe_2_(C_5_H_5_)_2_(C_14_H_19_BBrN)]
*M* *_r_* = 533.90
Orthorhombic, 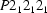

*a* = 8.8791 (2) Å
*b* = 9.2210 (2) Å
*c* = 27.1292 (6) Å
*V* = 2221.18 (9) Å^3^
*Z* = 4
Mo *K*α radiation
μ = 3.12 mm^−1^
*T* = 100 K
0.33 × 0.27 × 0.21 mm

#### Data collection

Bruker Kappa APEXII CCD diffractometer
Absorption correction: multi-scan (*SADABS*; Bruker, 2008 ▶) *T*_min_ = 0.54, *T*_max_ = 0.75
34891 measured reflections
6489 independent reflections
6288 reflections with *I* > 2σ(*I*)
*R*_int_ = 0.034

#### Refinement

*R*[*F* ^2^ > 2σ(*F* ^2^)] = 0.023
*wR*(*F* ^2^) = 0.054
*S* = 1.07
6489 reflections
266 parameters
H-atom parameters constrained
Δρ_max_ = 0.69 e Å^−3^
Δρ_min_ = −0.31 e Å^−3^
Absolute structure: Flack (1983 ▶), 2807 Friedel pairs
Flack parameter: 0.002 (5)

### 

Data collection: *APEX2* (Bruker, 2008 ▶); cell refinement: *SAINT* (Bruker, 2008 ▶); data reduction: *SAINT* and *XPREP* (Bruker, 2008 ▶); program(s) used to solve structure: *SHELXS97* (Sheldrick, 2008 ▶); program(s) used to refine structure: *SHELXL97* (Sheldrick, 2008 ▶); molecular graphics: *Mercury* (Macrae *et al.*, 2006 ▶); software used to prepare material for publication: *PLATON* (Spek, 2009 ▶) and *publCIF* (Westrip, 2010 ▶).

## Supplementary Material

Crystal structure: contains datablock(s) I, global. DOI: 10.1107/S1600536811049270/bq2322sup1.cif

Structure factors: contains datablock(s) I. DOI: 10.1107/S1600536811049270/bq2322Isup2.hkl

Additional supplementary materials:  crystallographic information; 3D view; checkCIF report

## Figures and Tables

**Table 1:** Hydrogen-bond geometry (Å, °)

*D*—H⋯*A*	*D*—H	H⋯*A*	*D*⋯*A*	*D*—H⋯*A*
C21—H21⋯Br1	1.00	2.79	3.7154 (17)	154

**Fig. 1.**
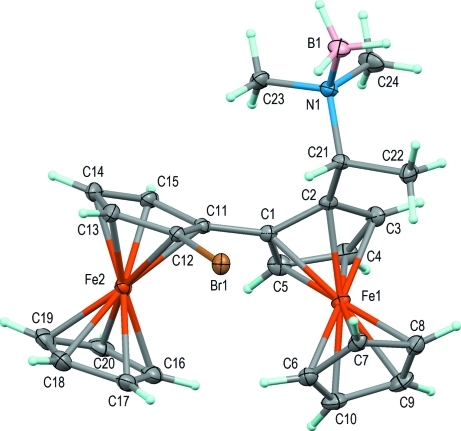
The asymmetric unit of the title compound with displacement ellipsoids for the non-hydrogen atoms drawn at the 50% probability level.

**Fig. 2.**
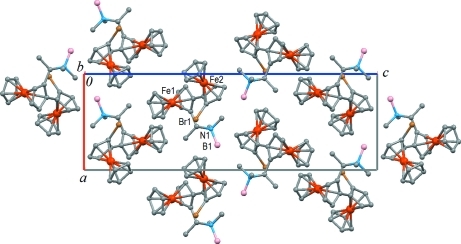
Crystal structure of the title compound with view along the crystallographic b-axis.

## supplementary crystallographic information

### Comment

Chiral non-racemic ferrocenyldiphosphines are widely used as ligands for highly enantioselective transition metal catalysts (Togni, 1996; Blaser *et al.*, 2007; Dai & Hou, 2010). Most of these ferrocenyldiphosphines are based on a planar chiral 1,2-disubstituted monoferrocene backbone. On the contrary, comparatively few chiral biferrocene derivatives are in use because they tend to be restricted to *C*_2_-symmetric entities like the BIFEP and TRAP biferrocene diphosphine ligands obtained by homocoupling of iodoferrocene derivatives, which curtails their modularity (Sawamura *et al.*, 1991; Nettekoven *et al.*, 2000; Xiao *et al.*, 2002; Espino *et al.*, 2009; Kuwano, 2010). Within a research program to open new synthetic pathways for asymmetrically substituted chiral non-racemic biferrocene diphosphines (Wang *et al.*, 2011) the title compound, (I), was obtained as an intermediate and studied by X-ray crystallography in order to fix its absolute configuration. A view of the asymmetric unit of (I) (Fig. 1) reveals that the compound has a planar-chiral *S*,*S*-configuration for the biferrocene fragment. Fe—C and ring C—C bond lengths show usual values (Fe—C = 2.015 (2) to 2.062 (2) Å, mean value 2.048 (10) Å; C—C = 1.417 (3) to 1.444 (3) Å, mean value 1.427 (8) Å) and both ferrocene groups have approximately staggered pairs of rings. The interplanar angle between the cyclopentadienyl rings C1 trough C5 (ring 1) and C11 through C15 (ring 3) is 54.29 (8)° and the torsion angle Fe1—*Cg*1—*Cg*3—Fe2 = -52.5° (*Cg*1 and 3 are the corresponding ring centroids). The dimethylamino and the BH_3_ group are *exo*-oriented displaying torsion angles of Fe1—C2—C21—N1 = 177.44 (12)° and C2—C21—N1—B1 = 176.91 (15)°. The bond lengths C12—Br1 = 1.8913 (19) Å and N1—B1 = 1.631 (1) Å adopt normal values, similar to a diastereomer of (I) (Wang *et al.*, 2011). The conformation of the molecule is stabilized by the intramolecular C21—H21···Br1 interaction with C···Br = 3.716 (2) Å. The arrangement and cohesion of the molecules in the crystal lattice (Fig. 2) is essentially based on unremarkable van-der-Waals interactions.

### Experimental

To a stirred solution of (*R*,*S*_Fc_,*S*_Fc_)-2''-bromo-2-[1-*N*,*N*-(dimethylamino)ethyl]-1,1''-biferrocene (100 mg, 0.19 mmol; Wang *et al.*, 2011) in THF (3 ml) was added BH_3_.THF (1 *M*, 0.7 ml, 0.7 mmol) at 0 °C. Stirring was continued for 1 h at r.t. before the reaction mixture was quenched at 0 °C by dropwise addition of water. The organics were extracted with dichloromethane, the combined solutions were washed with water and dried with MgSO_4_. The solvents were removed and the residue was purified by chromatography (aluminium oxide, eluent CH_2_Cl_2_). Crystals suitable for X-ray diffraction were obtained from ethyl acetate by slow evaporation at r.t..

^1^HNMR (400 MHz, CDCl_3_) δ 1.09–2.00 (br s, 3H, H1), 1.81 (s, 3H, H23), 1.84 (d, *J* = 6.9 Hz, 3H, H22), 2.13 (s, 3H, H24), 3.94 (dd, *J*_1_= 2.6 Hz, *J*_2_ = 1.6 Hz, 1H, H15), 4.05 (q, *J* = 6.9 Hz,1*H*, H21), 4.09 (dd, *J*_1_ = *J*_2_ = 2.6 Hz, 1H,H14), 4.36 (s, 5H, H16–H20), 4.36–4.38 (m, 1H, H3), 4.39 (s, 5H, H6–H10), 4.43(dd, *J*_1_ = *J*_2_ = 2.5 Hz, 1H, H4), 4.50 (dd, *J*_1_= 2.5 Hz, *J*_2_ = 1.5 Hz, 1H, H5), 4.64 (dd, *J*_1_= 2.6 Hz, *J*_2_ = 1.6 Hz, 1H, H13).

^13^C{^1^H} NMR (100.6 MHz, CDCl_3_):δ 17.8 (C22), 44.6 (C24), 52.5 (C23), 63.2 (C21), 66.5 (C14), 67.6(C4), 67.8 (C3), 69.3 (C5), 70.9 (5 C, C6–C10), 71.6 (5 C, C16–C20), 72.2 (C13),72.3 (C15).

### Refinement

All H atoms were placed in calculated positions and thereafter treated as riding, C—H = 0.95 – 1.00 Å, B—H = 0.98 Å. *U*_ĩso_(H) = 1.2*U*_eq_(C) for CH groups. *U*_ĩso_(H) = 1.5*U*_eq_(C,B) for CH_3_ and BH_3_ groups, which were refined with a torsional parameter.

### Crystal data

**Table:**

[Fe_2_(C_5_H_5_)_2_(C_14_H_19_BBrN)]	*F*(000) = 1088
*M**_r_* = 533.90	*D*_x_ = 1.597 Mg m^−^^3^
Orthorhombic, *P*2_1_2_1_2_1_	Mo *K*α radiation, λ = 0.71073 Å
Hall symbol: P 2ac 2ab	Cell parameters from 9916 reflections
*a* = 8.8791 (2) Å	θ = 2.3–30.1°
*b* = 9.2210 (2) Å	µ = 3.12 mm^−^^1^
*c* = 27.1292 (6) Å	*T* = 100 K
*V* = 2221.18 (9) Å^3^	Prism, yellow
*Z* = 4	0.33 × 0.27 × 0.21 mm

### Data collection

**Table:**

Bruker Kappa APEXII CCD diffractometer	6489 independent reflections
Radiation source: fine-focus sealed tube	6288 reflections with *I* > 2σ(*I*)
graphite	*R*_int_ = 0.034
φ and ω scans	θ_max_ = 30.1°, θ_min_ = 2.7°
Absorption correction: multi-scan (*SADABS*; Bruker, 2008)	*h* = −12→11
*T*_min_ = 0.54, *T*_max_ = 0.75	*k* = −12→12
34891 measured reflections	*l* = −38→38

### Refinement

**Table:**

Refinement on *F*^2^	Secondary atom site location: difference Fourier map
Least-squares matrix: full	Hydrogen site location: inferred from neighbouring sites
*R*[*F*^2^ > 2σ(*F*^2^)] = 0.023	H-atom parameters constrained
*wR*(*F*^2^) = 0.054	*w* = 1/[σ^2^(*F*_o_^2^) + (0.0234*P*)^2^ + 1.1731*P*] where *P* = (*F*_o_^2^ + 2*F*_c_^2^)/3
*S* = 1.07	(Δ/σ)_max_ = 0.001
6489 reflections	Δρ_max_ = 0.69 e Å^−^^3^
266 parameters	Δρ_min_ = −0.31 e Å^−^^3^
0 restraints	Absolute structure: Flack (1983), 2807 Friedel pairs
Primary atom site location: structure-invariant direct methods	Flack parameter: 0.002 (5)

### Special details

**Table:**

Geometry. All e.s.d.'s (except the e.s.d. in the dihedral angle between two l.s. planes) are estimated using the full covariance matrix. The cell e.s.d.'s are taken into account individually in the estimation of e.s.d.'s in distances, angles and torsion angles; correlations between e.s.d.'s in cell parameters are only used when they are defined by crystal symmetry. An approximate (isotropic) treatment of cell e.s.d.'s is used for estimating e.s.d.'s involving l.s. planes.
Refinement. Refinement of *F*^2^ against ALL reflections. The weighted *R*-factor *wR* and goodness of fit *S* are based on *F*^2^, conventional *R*-factors *R* are based on *F*, with *F* set to zero for negative *F*^2^. The threshold expression of *F*^2^ > σ(*F*^2^) is used only for calculating *R*-factors(gt) *etc*. and is not relevant to the choice of reflections for refinement. *R*-factors based on *F*^2^ are statistically about twice as large as those based on *F*, and *R*- factors based on ALL data will be even larger.

### Fractional atomic coordinates and isotropic or equivalent isotropic displacement parameters (Å^2^)

**Table:**

	*x*	*y*	*z*	*U*_iso_*/*U*_eq_	
Br1	0.47251 (2)	0.185812 (19)	0.380922 (6)	0.01470 (4)	
Fe1	0.30161 (3)	0.53063 (3)	0.298698 (9)	0.01275 (6)	
Fe2	0.08967 (3)	0.22678 (3)	0.408373 (10)	0.01436 (6)	
N1	0.57286 (19)	0.66124 (17)	0.43320 (6)	0.0148 (3)	
B1	0.7399 (3)	0.6249 (3)	0.45489 (8)	0.0203 (4)	
H1A	0.7446	0.6526	0.4897	0.030*	
H1B	0.8157	0.6795	0.4363	0.030*	
H1C	0.7598	0.5208	0.4517	0.030*	
C1	0.2564 (2)	0.5202 (2)	0.37276 (6)	0.0123 (3)	
C2	0.3899 (2)	0.60595 (19)	0.36404 (6)	0.0120 (3)	
C3	0.3469 (2)	0.7240 (2)	0.33252 (7)	0.0162 (4)	
H3	0.4123	0.7975	0.3205	0.019*	
C4	0.1907 (3)	0.7127 (2)	0.32241 (7)	0.0199 (4)	
H4	0.1337	0.7774	0.3026	0.024*	
C5	0.1340 (2)	0.5881 (2)	0.34696 (7)	0.0176 (4)	
H5	0.0326	0.5554	0.3464	0.021*	
C6	0.2974 (2)	0.3255 (2)	0.27028 (7)	0.0178 (3)	
H6	0.2661	0.2406	0.2873	0.021*	
C7	0.4464 (2)	0.3820 (2)	0.26885 (6)	0.0159 (4)	
H7	0.5320	0.3414	0.2847	0.019*	
C8	0.4446 (2)	0.5103 (2)	0.23954 (7)	0.0180 (4)	
H8	0.5288	0.5704	0.2325	0.022*	
C9	0.2947 (3)	0.5326 (2)	0.22272 (7)	0.0227 (4)	
H9	0.2612	0.6100	0.2024	0.027*	
C10	0.2035 (3)	0.4183 (2)	0.24179 (7)	0.0224 (4)	
H10	0.0985	0.4063	0.2364	0.027*	
C11	0.2337 (2)	0.4012 (2)	0.40848 (7)	0.0125 (3)	
C12	0.3112 (2)	0.2670 (2)	0.41713 (6)	0.0134 (3)	
C13	0.2534 (2)	0.1987 (2)	0.46023 (7)	0.0180 (4)	
H13	0.2861	0.1093	0.4739	0.022*	
C14	0.1379 (2)	0.2896 (2)	0.47874 (7)	0.0198 (4)	
H14	0.0789	0.2713	0.5073	0.024*	
C15	0.1248 (2)	0.4126 (2)	0.44759 (7)	0.0180 (4)	
H15	0.0554	0.4900	0.4519	0.022*	
C16	−0.0151 (2)	0.2224 (2)	0.34155 (7)	0.0210 (4)	
H16	0.0022	0.2875	0.3150	0.025*	
C17	0.0622 (2)	0.0887 (2)	0.35010 (7)	0.0202 (4)	
H17	0.1403	0.0495	0.3302	0.024*	
C18	0.0020 (3)	0.0248 (2)	0.39343 (7)	0.0233 (4)	
H18	0.0326	−0.0647	0.4076	0.028*	
C19	−0.1121 (3)	0.1181 (2)	0.41198 (9)	0.0255 (4)	
H19	−0.1709	0.1015	0.4407	0.031*	
C20	−0.1235 (2)	0.2405 (2)	0.38022 (8)	0.0229 (4)	
H20	−0.1907	0.3198	0.3840	0.028*	
C21	0.5465 (2)	0.58227 (18)	0.38436 (6)	0.0122 (3)	
H21	0.5562	0.4762	0.3913	0.015*	
C22	0.6685 (2)	0.6203 (2)	0.34687 (7)	0.0192 (4)	
H22A	0.6581	0.5578	0.3178	0.029*	
H22B	0.7679	0.6055	0.3617	0.029*	
H22C	0.6578	0.7220	0.3370	0.029*	
C23	0.4604 (3)	0.6085 (2)	0.46983 (7)	0.0190 (4)	
H23A	0.4772	0.6570	0.5015	0.029*	
H23B	0.4714	0.5035	0.4741	0.029*	
H23C	0.3586	0.6303	0.4580	0.029*	
C24	0.5543 (3)	0.8216 (2)	0.42826 (8)	0.0235 (4)	
H24A	0.4509	0.8435	0.4181	0.035*	
H24B	0.6248	0.8583	0.4034	0.035*	
H24C	0.5750	0.8681	0.4600	0.035*	

### Atomic displacement parameters (Å^2^)

**Table:**

	*U*^11^	*U*^22^	*U*^33^	*U*^12^	*U*^13^	*U*^23^
Br1	0.01301 (8)	0.01403 (7)	0.01707 (8)	0.00257 (7)	0.00186 (7)	0.00236 (6)
Fe1	0.01316 (13)	0.01367 (12)	0.01141 (11)	0.00278 (10)	−0.00227 (10)	0.00025 (9)
Fe2	0.01111 (13)	0.01697 (12)	0.01499 (12)	−0.00394 (10)	0.00234 (10)	−0.00310 (9)
N1	0.0172 (8)	0.0131 (7)	0.0141 (7)	−0.0022 (6)	−0.0019 (6)	−0.0017 (5)
B1	0.0170 (11)	0.0248 (11)	0.0191 (10)	−0.0028 (9)	−0.0051 (8)	−0.0003 (8)
C1	0.0099 (8)	0.0141 (8)	0.0129 (7)	0.0009 (6)	0.0002 (6)	−0.0022 (6)
C2	0.0117 (9)	0.0119 (7)	0.0124 (7)	0.0018 (6)	0.0002 (6)	−0.0024 (6)
C3	0.0192 (10)	0.0123 (8)	0.0171 (8)	0.0023 (7)	−0.0013 (7)	0.0010 (7)
C4	0.0207 (10)	0.0171 (9)	0.0219 (9)	0.0077 (8)	−0.0032 (8)	−0.0009 (7)
C5	0.0114 (9)	0.0200 (9)	0.0215 (9)	0.0053 (7)	−0.0011 (7)	−0.0026 (7)
C6	0.0195 (9)	0.0180 (8)	0.0159 (8)	−0.0017 (8)	0.0012 (7)	−0.0030 (7)
C7	0.0195 (11)	0.0152 (8)	0.0130 (7)	0.0032 (7)	0.0008 (6)	−0.0008 (6)
C8	0.0216 (11)	0.0180 (9)	0.0143 (8)	0.0009 (7)	0.0022 (7)	0.0013 (6)
C9	0.0277 (11)	0.0277 (10)	0.0129 (8)	0.0057 (9)	−0.0038 (8)	0.0020 (7)
C10	0.0208 (10)	0.0297 (11)	0.0167 (8)	0.0007 (9)	−0.0060 (8)	−0.0058 (8)
C11	0.0096 (8)	0.0156 (8)	0.0124 (7)	−0.0027 (6)	0.0005 (6)	−0.0028 (6)
C12	0.0120 (9)	0.0168 (8)	0.0115 (7)	−0.0035 (7)	0.0001 (6)	−0.0012 (6)
C13	0.0187 (10)	0.0215 (9)	0.0137 (7)	−0.0048 (8)	−0.0007 (6)	0.0024 (7)
C14	0.0167 (9)	0.0278 (11)	0.0149 (8)	−0.0087 (8)	0.0040 (7)	−0.0027 (7)
C15	0.0155 (10)	0.0231 (9)	0.0153 (8)	−0.0042 (7)	0.0035 (7)	−0.0060 (7)
C16	0.0174 (10)	0.0248 (9)	0.0207 (8)	−0.0060 (8)	−0.0014 (7)	−0.0052 (7)
C17	0.0190 (11)	0.0208 (9)	0.0209 (9)	−0.0056 (7)	0.0016 (7)	−0.0084 (7)
C18	0.0265 (12)	0.0200 (9)	0.0235 (9)	−0.0089 (8)	0.0011 (8)	−0.0031 (7)
C19	0.0180 (11)	0.0299 (11)	0.0285 (10)	−0.0116 (9)	0.0031 (8)	−0.0057 (9)
C20	0.0124 (9)	0.0283 (10)	0.0281 (10)	−0.0036 (7)	−0.0025 (8)	−0.0095 (9)
C21	0.0111 (8)	0.0127 (7)	0.0128 (7)	−0.0003 (6)	−0.0011 (6)	−0.0022 (6)
C22	0.0147 (10)	0.0272 (10)	0.0158 (8)	−0.0035 (8)	0.0026 (7)	0.0005 (7)
C23	0.0201 (10)	0.0230 (9)	0.0140 (8)	−0.0023 (8)	0.0036 (7)	−0.0044 (7)
C24	0.0306 (12)	0.0117 (8)	0.0283 (10)	0.0005 (8)	−0.0067 (8)	−0.0028 (7)

### Geometric parameters (Å, °)

**Table:**

Br1—C12	1.8913 (19)	C6—C10	1.423 (3)
Fe1—C6	2.043 (2)	C6—H6	0.9500
Fe1—C3	2.046 (2)	C7—C8	1.426 (3)
Fe1—C7	2.046 (2)	C7—H7	0.9500
Fe1—C4	2.050 (2)	C8—C9	1.421 (3)
Fe1—C1	2.051 (2)	C8—H8	0.9500
Fe1—C5	2.052 (2)	C9—C10	1.426 (3)
Fe1—C10	2.053 (2)	C9—H9	0.9500
Fe1—C8	2.055 (2)	C10—H10	0.9500
Fe1—C2	2.059 (2)	C11—C12	1.435 (3)
Fe1—C9	2.062 (2)	C11—C15	1.440 (3)
Fe2—C12	2.015 (2)	C12—C13	1.424 (2)
Fe2—C16	2.038 (2)	C13—C14	1.417 (3)
Fe2—C13	2.040 (2)	C13—H13	0.9500
Fe2—C14	2.041 (2)	C14—C15	1.419 (3)
Fe2—C15	2.041 (2)	C14—H14	0.9500
Fe2—C17	2.044 (2)	C15—H15	0.9500
Fe2—C20	2.045 (2)	C16—C17	1.430 (3)
Fe2—C11	2.055 (2)	C16—C20	1.433 (3)
Fe2—C19	2.056 (2)	C16—H16	0.9500
Fe2—C18	2.059 (2)	C17—C18	1.419 (3)
N1—C23	1.490 (3)	C17—H17	0.9500
N1—C24	1.494 (3)	C18—C19	1.421 (3)
N1—C21	1.530 (2)	C18—H18	0.9500
N1—B1	1.631 (3)	C19—C20	1.424 (3)
B1—H1A	0.9800	C19—H19	0.9500
B1—H1B	0.9800	C20—H20	0.9500
B1—H1C	0.9800	C21—C22	1.527 (3)
C1—C5	1.437 (3)	C21—H21	1.0000
C1—C2	1.444 (3)	C22—H22A	0.9800
C1—C11	1.477 (3)	C22—H22B	0.9800
C2—C3	1.436 (2)	C22—H22C	0.9800
C2—C21	1.512 (3)	C23—H23A	0.9800
C3—C4	1.418 (3)	C23—H23B	0.9800
C3—H3	0.9500	C23—H23C	0.9800
C4—C5	1.420 (3)	C24—H24A	0.9800
C4—H4	0.9500	C24—H24B	0.9800
C5—H5	0.9500	C24—H24C	0.9800
C6—C7	1.422 (3)		
			
C6—Fe1—C3	168.54 (8)	C3—C4—H4	125.8
C6—Fe1—C7	40.70 (8)	C5—C4—H4	125.8
C3—Fe1—C7	129.63 (8)	Fe1—C4—H4	126.4
C6—Fe1—C4	150.24 (9)	C4—C5—C1	108.25 (18)
C3—Fe1—C4	40.51 (8)	C4—C5—Fe1	69.66 (12)
C7—Fe1—C4	167.05 (8)	C1—C5—Fe1	69.48 (11)
C6—Fe1—C1	108.81 (7)	C4—C5—H5	125.9
C3—Fe1—C1	68.91 (7)	C1—C5—H5	125.9
C7—Fe1—C1	118.62 (7)	Fe1—C5—H5	126.6
C4—Fe1—C1	68.73 (8)	C7—C6—C10	108.03 (18)
C6—Fe1—C5	117.84 (8)	C7—C6—Fe1	69.75 (11)
C3—Fe1—C5	68.35 (8)	C10—C6—Fe1	70.03 (12)
C7—Fe1—C5	151.80 (8)	C7—C6—H6	126.0
C4—Fe1—C5	40.51 (8)	C10—C6—H6	126.0
C1—Fe1—C5	40.99 (8)	Fe1—C6—H6	125.8
C6—Fe1—C10	40.67 (8)	C6—C7—C8	107.99 (17)
C3—Fe1—C10	149.38 (8)	C6—C7—Fe1	69.55 (11)
C7—Fe1—C10	68.35 (9)	C8—C7—Fe1	69.99 (11)
C4—Fe1—C10	116.45 (9)	C6—C7—H7	126.0
C1—Fe1—C10	129.05 (8)	C8—C7—H7	126.0
C5—Fe1—C10	107.62 (9)	Fe1—C7—H7	126.0
C6—Fe1—C8	68.41 (8)	C9—C8—C7	108.04 (18)
C3—Fe1—C8	107.95 (8)	C9—C8—Fe1	70.08 (11)
C7—Fe1—C8	40.69 (7)	C7—C8—Fe1	69.32 (11)
C4—Fe1—C8	128.05 (8)	C9—C8—H8	126.0
C1—Fe1—C8	151.85 (8)	C7—C8—H8	126.0
C5—Fe1—C8	165.93 (8)	Fe1—C8—H8	126.2
C10—Fe1—C8	68.20 (9)	C8—C9—C10	107.95 (18)
C6—Fe1—C2	130.10 (7)	C8—C9—Fe1	69.53 (11)
C3—Fe1—C2	40.96 (7)	C10—C9—Fe1	69.37 (11)
C7—Fe1—C2	109.12 (7)	C8—C9—H9	126.0
C4—Fe1—C2	68.67 (8)	C10—C9—H9	126.0
C1—Fe1—C2	41.14 (7)	Fe1—C9—H9	126.6
C5—Fe1—C2	68.86 (8)	C6—C10—C9	108.0 (2)
C10—Fe1—C2	168.28 (8)	C6—C10—Fe1	69.30 (11)
C8—Fe1—C2	117.91 (8)	C9—C10—Fe1	70.07 (12)
C6—Fe1—C9	68.32 (8)	C6—C10—H10	126.0
C3—Fe1—C9	116.51 (8)	C9—C10—H10	126.0
C7—Fe1—C9	68.23 (8)	Fe1—C10—H10	126.2
C4—Fe1—C9	106.98 (8)	C12—C11—C15	105.34 (16)
C1—Fe1—C9	166.83 (9)	C12—C11—C1	133.01 (16)
C5—Fe1—C9	127.89 (9)	C15—C11—C1	121.38 (17)
C10—Fe1—C9	40.56 (9)	C12—C11—Fe2	67.89 (10)
C8—Fe1—C9	40.39 (9)	C15—C11—Fe2	68.91 (11)
C2—Fe1—C9	150.32 (9)	C1—C11—Fe2	131.69 (13)
C12—Fe2—C16	123.64 (8)	C13—C12—C11	110.07 (17)
C12—Fe2—C13	41.10 (7)	C13—C12—Br1	121.62 (15)
C16—Fe2—C13	159.46 (8)	C11—C12—Br1	128.27 (13)
C12—Fe2—C14	68.45 (8)	C13—C12—Fe2	70.36 (11)
C16—Fe2—C14	159.00 (9)	C11—C12—Fe2	70.83 (11)
C13—Fe2—C14	40.64 (8)	Br1—C12—Fe2	127.24 (9)
C12—Fe2—C15	68.59 (8)	C14—C13—C12	106.86 (18)
C16—Fe2—C15	123.37 (9)	C14—C13—Fe2	69.71 (11)
C13—Fe2—C15	68.79 (9)	C12—C13—Fe2	68.54 (11)
C14—Fe2—C15	40.69 (8)	C14—C13—H13	126.6
C12—Fe2—C17	108.80 (8)	C12—C13—H13	126.6
C16—Fe2—C17	41.00 (9)	Fe2—C13—H13	126.7
C13—Fe2—C17	122.65 (9)	C13—C14—C15	108.74 (17)
C14—Fe2—C17	157.73 (9)	C13—C14—Fe2	69.64 (11)
C15—Fe2—C17	160.77 (8)	C15—C14—Fe2	69.68 (11)
C12—Fe2—C20	159.37 (8)	C13—C14—H14	125.6
C16—Fe2—C20	41.10 (9)	C15—C14—H14	125.6
C13—Fe2—C20	157.70 (8)	Fe2—C14—H14	126.6
C14—Fe2—C20	121.74 (9)	C14—C15—C11	108.99 (18)
C15—Fe2—C20	106.51 (8)	C14—C15—Fe2	69.63 (11)
C17—Fe2—C20	68.84 (9)	C11—C15—Fe2	69.93 (10)
C12—Fe2—C11	41.28 (7)	C14—C15—H15	125.5
C16—Fe2—C11	107.51 (8)	C11—C15—H15	125.5
C13—Fe2—C11	69.80 (8)	Fe2—C15—H15	126.5
C14—Fe2—C11	69.25 (8)	C17—C16—C20	107.67 (18)
C15—Fe2—C11	41.15 (7)	C17—C16—Fe2	69.74 (11)
C17—Fe2—C11	124.25 (8)	C20—C16—Fe2	69.69 (11)
C20—Fe2—C11	121.89 (8)	C17—C16—H16	126.2
C12—Fe2—C19	159.19 (9)	C20—C16—H16	126.2
C16—Fe2—C19	68.58 (9)	Fe2—C16—H16	126.0
C13—Fe2—C19	121.78 (9)	C18—C17—C16	108.16 (19)
C14—Fe2—C19	106.07 (9)	C18—C17—Fe2	70.32 (11)
C15—Fe2—C19	121.18 (9)	C16—C17—Fe2	69.27 (11)
C17—Fe2—C19	68.22 (9)	C18—C17—H17	125.9
C20—Fe2—C19	40.63 (9)	C16—C17—H17	125.9
C11—Fe2—C19	157.55 (9)	Fe2—C17—H17	126.1
C12—Fe2—C18	123.97 (9)	C17—C18—C19	108.1 (2)
C16—Fe2—C18	68.54 (9)	C17—C18—Fe2	69.21 (11)
C13—Fe2—C18	106.89 (9)	C19—C18—Fe2	69.67 (12)
C14—Fe2—C18	121.37 (8)	C17—C18—H18	126.0
C15—Fe2—C18	156.91 (8)	C19—C18—H18	126.0
C17—Fe2—C18	40.47 (8)	Fe2—C18—H18	126.7
C20—Fe2—C18	68.42 (9)	C18—C19—C20	108.41 (19)
C11—Fe2—C18	160.67 (8)	C18—C19—Fe2	69.92 (12)
C19—Fe2—C18	40.41 (9)	C20—C19—Fe2	69.27 (12)
C23—N1—C24	107.98 (16)	C18—C19—H19	125.8
C23—N1—C21	108.64 (14)	C20—C19—H19	125.8
C24—N1—C21	112.12 (14)	Fe2—C19—H19	126.6
C23—N1—B1	107.57 (15)	C19—C20—C16	107.67 (19)
C24—N1—B1	109.64 (16)	C19—C20—Fe2	70.10 (13)
C21—N1—B1	110.73 (15)	C16—C20—Fe2	69.21 (11)
N1—B1—H1A	109.5	C19—C20—H20	126.2
N1—B1—H1B	109.5	C16—C20—H20	126.2
H1A—B1—H1B	109.5	Fe2—C20—H20	126.1
N1—B1—H1C	109.5	C2—C21—C22	112.12 (15)
H1A—B1—H1C	109.5	C2—C21—N1	112.82 (14)
H1B—B1—H1C	109.5	C22—C21—N1	111.04 (15)
C5—C1—C2	107.56 (16)	C2—C21—H21	106.8
C5—C1—C11	122.70 (17)	C22—C21—H21	106.8
C2—C1—C11	128.77 (16)	N1—C21—H21	106.8
C5—C1—Fe1	69.53 (11)	C21—C22—H22A	109.5
C2—C1—Fe1	69.71 (10)	C21—C22—H22B	109.5
C11—C1—Fe1	134.76 (13)	H22A—C22—H22B	109.5
C3—C2—C1	107.17 (16)	C21—C22—H22C	109.5
C3—C2—C21	124.81 (17)	H22A—C22—H22C	109.5
C1—C2—C21	128.00 (15)	H22B—C22—H22C	109.5
C3—C2—Fe1	69.03 (10)	N1—C23—H23A	109.5
C1—C2—Fe1	69.15 (10)	N1—C23—H23B	109.5
C21—C2—Fe1	127.99 (12)	H23A—C23—H23B	109.5
C4—C3—C2	108.60 (18)	N1—C23—H23C	109.5
C4—C3—Fe1	69.90 (11)	H23A—C23—H23C	109.5
C2—C3—Fe1	70.01 (10)	H23B—C23—H23C	109.5
C4—C3—H3	125.7	N1—C24—H24A	109.5
C2—C3—H3	125.7	N1—C24—H24B	109.5
Fe1—C3—H3	126.0	H24A—C24—H24B	109.5
C3—C4—C5	108.41 (17)	N1—C24—H24C	109.5
C3—C4—Fe1	69.59 (11)	H24A—C24—H24C	109.5
C5—C4—Fe1	69.82 (11)	H24B—C24—H24C	109.5
			
C5—C1—C2—C3	−0.7 (2)	Fe2—C11—C12—C13	−59.61 (13)
C11—C1—C2—C3	−169.48 (17)	C15—C11—C12—Br1	−178.14 (14)
C5—C1—C2—C21	177.98 (17)	C1—C11—C12—Br1	−4.3 (3)
C11—C1—C2—C21	9.2 (3)	Fe2—C11—C12—Br1	122.80 (15)
Fe1—C1—C2—C21	−122.56 (18)	C15—C11—C12—Fe2	59.06 (12)
C5—C1—C2—Fe1	−59.46 (12)	C1—C11—C12—Fe2	−127.1 (2)
C11—C1—C2—Fe1	131.75 (19)	C11—C12—C13—C14	0.5 (2)
C1—C2—C3—C4	0.6 (2)	Br1—C12—C13—C14	178.23 (13)
C21—C2—C3—C4	−178.11 (16)	Fe2—C12—C13—C14	−59.44 (13)
Fe1—C2—C3—C4	59.45 (13)	C11—C12—C13—Fe2	59.90 (13)
C1—C2—C3—Fe1	−58.84 (12)	Br1—C12—C13—Fe2	−122.33 (13)
C21—C2—C3—Fe1	122.43 (17)	C12—C13—C14—C15	−0.2 (2)
C2—C3—C4—C5	−0.3 (2)	Fe2—C13—C14—C15	−58.87 (14)
Fe1—C3—C4—C5	59.22 (14)	C12—C13—C14—Fe2	58.69 (13)
C2—C3—C4—Fe1	−59.52 (13)	C13—C14—C15—C11	−0.2 (2)
C3—C4—C5—C1	−0.1 (2)	Fe2—C14—C15—C11	−59.02 (13)
Fe1—C4—C5—C1	58.94 (13)	C13—C14—C15—Fe2	58.84 (14)
C3—C4—C5—Fe1	−59.08 (14)	C12—C11—C15—C14	0.4 (2)
C2—C1—C5—C4	0.5 (2)	C1—C11—C15—C14	−174.31 (16)
C11—C1—C5—C4	170.14 (16)	Fe2—C11—C15—C14	58.83 (14)
Fe1—C1—C5—C4	−59.06 (13)	C12—C11—C15—Fe2	−58.39 (12)
C2—C1—C5—Fe1	59.57 (12)	C1—C11—C15—Fe2	126.86 (17)
C11—C1—C5—Fe1	−130.80 (17)	C20—C16—C17—C18	0.2 (2)
C10—C6—C7—C8	0.2 (2)	Fe2—C16—C17—C18	59.79 (14)
Fe1—C6—C7—C8	−59.66 (13)	C20—C16—C17—Fe2	−59.61 (13)
C10—C6—C7—Fe1	59.81 (14)	C16—C17—C18—C19	−0.1 (2)
C6—C7—C8—C9	−0.2 (2)	Fe2—C17—C18—C19	59.00 (15)
Fe1—C7—C8—C9	−59.61 (14)	C16—C17—C18—Fe2	−59.13 (14)
C6—C7—C8—Fe1	59.38 (13)	C17—C18—C19—C20	0.0 (2)
C7—C8—C9—C10	0.2 (2)	Fe2—C18—C19—C20	58.75 (14)
Fe1—C8—C9—C10	−58.92 (14)	C17—C18—C19—Fe2	−58.72 (14)
C7—C8—C9—Fe1	59.13 (13)	C18—C19—C20—C16	0.1 (2)
C7—C6—C10—C9	0.0 (2)	Fe2—C19—C20—C16	59.23 (14)
Fe1—C6—C10—C9	59.62 (14)	C18—C19—C20—Fe2	−59.15 (15)
C7—C6—C10—Fe1	−59.64 (13)	C17—C16—C20—C19	−0.2 (2)
C8—C9—C10—C6	−0.1 (2)	Fe2—C16—C20—C19	−59.80 (14)
Fe1—C9—C10—C6	−59.14 (13)	C17—C16—C20—Fe2	59.63 (14)
C8—C9—C10—Fe1	59.02 (14)	C3—C2—C21—C22	−38.2 (2)
C5—C1—C11—C12	137.0 (2)	C1—C2—C21—C22	143.31 (18)
C2—C1—C11—C12	−55.7 (3)	Fe1—C2—C21—C22	51.2 (2)
Fe1—C1—C11—C12	44.1 (3)	C3—C2—C21—N1	88.0 (2)
C5—C1—C11—C15	−49.9 (3)	C1—C2—C21—N1	−90.4 (2)
C2—C1—C11—C15	117.3 (2)	Fe1—C2—C21—N1	177.44 (12)
Fe1—C1—C11—C15	−142.88 (17)	C23—N1—C21—C2	58.98 (19)
C5—C1—C11—Fe2	38.9 (3)	C24—N1—C21—C2	−60.3 (2)
C2—C1—C11—Fe2	−153.86 (15)	B1—N1—C21—C2	176.91 (15)
Fe1—C1—C11—Fe2	−54.1 (3)	C23—N1—C21—C22	−174.18 (16)
C15—C11—C12—C13	−0.6 (2)	C24—N1—C21—C22	66.6 (2)
C1—C11—C12—C13	173.31 (19)	B1—N1—C21—C22	−56.26 (19)

### Hydrogen-bond geometry (Å, °)

**Table:**

*D*—H···*A*	*D*—H	H···*A*	*D*···*A*	*D*—H···*A*
C21—H21···Br1	1.00	2.79	3.7154 (17)	154.
